# Training in experimental design and statistics is essential: Response to Jordan

**DOI:** 10.1371/journal.pbio.3000022

**Published:** 2018-10-15

**Authors:** Stanley E. Lazic, Charlie J. Clarke-Williams, Marcus R. Munafò

**Affiliations:** 1 Quantitative Biology, Discovery Sciences, IMED Biotech Unit, AstraZeneca, Cambridge, United Kingdom; 2 School of Physiology, Pharmacology and Neuroscience, University of Bristol, Bristol, United Kingdom; 3 MRC Integrative Epidemiology Unit, School of Experimental Psychology, University of Bristol, Bristol, United Kingdom

## Abstract

This Formal Comment responds to Jordan et al., and stresses that if scientific findings are to be robust, training in experimental design and statistics is critical to ensure that research questions, design considerations, and analyses are aligned.

The Formal Comment by Jordan [1] provides a valuable extension of the issues we discuss in our original article and provides us with an opportunity to clarify our arguments. Our discussion on pseudoreplication focused on defining the appropriate units to replicate when designing an experiment. Analysis of the resulting designs was beyond the scope of the paper, as it is a large topic, and we provide detailed examples elsewhere [2]. Below, we elaborate on aspects that may have been unclear.

The example in Figure 2C of the paper should indeed be analysed with a paired *t* test (or more generally, ‘animal’ should be included as a blocking variable in the model, which also addresses the ‘body parts’ example), as Jordan argues and as we also recommend [2, p. 120]. In retrospect, we could have mentioned this.

Confusion in example Figure 2D might have arisen by assuming that the experiment has multiple mice, and we only illustrated one for simplicity. In this example, the single mouse is the whole experiment, and a valid *p*-value can be calculated for the treatment effect using an independent samples *t* test (assuming there are no carryover effects, time trends, or other such complications). Each measurement is independent (technically, the measurements are conditionally independent given the mouse, and there is only one mouse, so the measurements are independent). N is the number of times the animal was randomised to a treatment condition, which equals the number of measurements in this example. This classic *N*-of-1 design is rarely used in experimental biology, but Edgington and Onghena provide an excellent introduction [3, Ch. 11]. An *N*-of-1 experiment enables one to ask the question ‘Does the treatment have an effect in this mouse?’, which would only make sense if this mouse is the biological unit of interest, for example, if it is your pet mouse and you are interested in the effect of two types of environments on some aspect of its behaviour. However, most researchers would be interested in establishing that the effect holds for mice in general, so multiple mice are needed, and the points made by Jordan then apply (that is, the multiple measurements on each animal do not contribute to N, and this needs to be accounted for in the analysis).

Jordan also comments on how nonindependence can arise from how a population is sampled. It may not be obvious, but this situation is covered in Figure 3 (and in [2, p. 60] and [4]) in which the litters represent different populations or (using our terminology) ‘recognisable subgroups’ and how they relate to the treatment effects of interest. We consciously avoided talking about ‘sampling from populations’ because laboratory-based biological research rarely involves sampling (subsampling is more common [2, p. 72]), and statistical tests are not justified by random sampling but by random assignment [3]. Biologists are often taught that statistics is the process of making inferences from samples to populations, which unfortunately does not reflect the majority of biomedical research but is appropriate for a survey or opinion poll. The samples-to-populations approach also does not encourage scientists to explicitly define the experimental unit, compared with teaching inference from a randomisation or permutation perspective [3]. We agree with Edgington and Onghena that ‘ …statistical inferences about populations are usually irrelevant …there is no logical connection between the random sampling model and its application to data from the typical experiment’ [3, Preface]. However, we acknowledge that sampling from populations is more common in ecology, evolution, or fisheries research for which experiments are conducted in the field.

We also agree that the analysis should follow the design and that the design should reflect the research question. However, we disagree with Jordan’s closing comment that pseudoreplication can be avoided by simply considering the sampling, design, research question, and analysis. While this is true in principle, in practice, it is not so straightforward. Without a strong foundation in the design of experiments and in data analysis, additional contemplation is unlikely to improve an experiment on its own. Hence, we argue for greater training in experimental design and statistics, which has the additional advantage of improving reproducibility more widely [2]. Only in this way will research questions, design considerations, and analytical approaches be aligned, and the conclusions drawn are more likely to be robust.
